# Differential expression of Ago2‐mediated microRNA signaling in adipose tissue is associated with food‐induced obesity

**DOI:** 10.1002/2211-5463.13471

**Published:** 2022-09-05

**Authors:** Hansi Zhang, Liang Qiao, Xiaoxuan Liu, Xiaojing Han, Jing Kang, Yanli Liu, Juntang Lin, Xin Yan

**Affiliations:** ^1^ School of Life Science and Technology Xinxiang Medical University China; ^2^ Stem Cell and Biotherapy Technology Research Center Xinxiang Medical University China; ^3^ Henan Joint International Research Laboratory of Stem Cell Medicine Xinxiang Medical University China

**Keywords:** adipose tissue, energy homeostasis, microRNAs, obesity, RNA‐binding proteins

## Abstract

Adipose tissue is a major component for the regulation of energy homeostasis by storage and release of lipids. As a core element of RNA‐induced silencing complex, argonaute2 (Ago2) plays critical role in maintenance of systemic metabolic demand. Here, we show that high‐fat‐diet‐fed mice exhibit an increase in body mass alongside systematic insulin resistance and altered rate of energy expenditure. Interestingly, *Ago2* expression is associated with obesity and an increased amount of adipose tissue. Moreover, increased levels of Ago2 inhibited the expression of AMPKα by promoting its targeting by *miR‐148a*, the most abundant microRNA in adipose tissues. Those results suggested that Ago2–*miR‐148a*–*AMPKα* signaling pathway play an important function in the developing obesity and adiposity, and will further provide basic research data for the potential clinical treatment of obesity.

## Graphical abstract

Here, we show that Ago2 expression is increased in adipose tissue of mice with food‐induced obesity. An increase in Ago2 promotes the expression of microRNA‐148a and subsequently inhibits the expression of its target AMPKα, a regulator of insulin sensitivity and energy homeostasis in adipose tissue. This study identifies that the Ago2‐miR‐148a signaling pathway plays a key role in the progression of diet‐induced obesity.

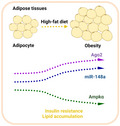

**Abbreviations**
ACC1: acetyl‐Coenzyme A carboxylase alpha
ACC2: acetyl‐Coenzyme A carboxylase beta
Ago2: argonaute 2
AMPKα1: protein kinase, AMP‐activated, alpha 1 catalytic subunit
BAT: Brown adipose tissue
Cd36: CD36 molecule
Chow: chow diet
ChREBP: MLX interacting protein‐like
Cidea: cell death‐inducing DNA fragmentation factor, alpha subunit‐like effector A
Cox8b: cytochrome *c* oxidase subunit 8B
Cpt1: carnitine palmitoyltransferase 1a
EE: energy expenditure
ELISA: enzyme‐linked immunosorbent assay
Elovl3: elongation of very long chain fatty acids
eWAT: epididymal white adipose tissue
Fas: fatty acid synthase
FFA: free fat acid
GPAT: glycerol‐3‐phosphate acyltransferase, mitochondrial
GTT: glucose tolerance test
HFD: high‐fat diet
HMGCR: 3‐hydroxy‐3‐methylglutaryl‐Coenzyme A reductase
ingWAT: inguinal white adipose tissue
ITT: insulin tolerance test
LBM: lean body mass
Ldlr: low density lipoprotein receptor
miRNA: microRNA
RER: respiratory exchange ratio
RISC: RNA‐induced silencing complex
S14: SPOT14, thyroid hormone responsive
Srebp1: sterol regulatory element binding transcription factor 1
sWAT: subcutaneous white adipose tissue
TD‐NMR: time‐domain nuclear magnetic resonance
Ucp1: uncoupling protein 1
Ucp2: uncoupling protein 2

Obesity is a chronic progressive disease and characterized by energy imbalance resulting from excessive calorie intake and abnormal energy expenditure [1] Generally, it is associated with type 2 diabetes, insulin resistance, and inflammation [2, 3]. As the prevalence of the disease continues to increase worldwide, it remains imperative to identify novel genes and their pathways that contribute to obesity. Recent studies have shown that several tissue‐specific microRNAs (miRNA) contributed to the progression of obesity [4, 5], dissecting their mechanism of action *in vivo* remains a challenge.

Argonaute2 (Ago2) is a core component of RNA‐induced silencing complex (RISC) and can mediate miRNA to interact with target mRNA [6, 7]. Interestingly, Ago2‐related miRNA pathway is emerging as an important contributor in several tissues and essential to regulate glucose metabolism and cellular energy homeostasis [8, 9, 10, 11]. For example, Ago2 is considered to be an important regulator of liver energy metabolism and high‐fat diet feeding forces liver to express excessive Ago2 in liver [10]. Conditional deletion of Ago2 in liver can improve the glucose metabolism through the activation of AMPK signaling pathway during high‐fat diet (HFD) feeding [10, 11]. Furthermore, obesity significantly increases the expression of Ago2 in islets, giving rise to pancreatic β‐cell proliferation and insulin resistance, which further provide evidence for Ago2 in the regulation of cellular metabolism [8].

Adipose tissues have an important function in sensing and managing energy status through lipid storage and release, and adipokines secretion [12, 13]. Strongly, accumulation of excess visceral adiposity can cause obesity which is associated with different metabolic dysfunction including chronic inflammation [13, 14]. Several studies have reported that many miRNAs in adipose tissues including *miR‐34a*, *miR‐93*, *miR‐106*, *miR‐148a*, and *miR‐221* have been shown to affect adipogenesis and adiposity [4, 15, 16, 17, 18]. For example, *miR‐93* has been shown to control adipocyte differentiation by negatively regulating *Tbx3*, and loss of *miR‐93* results in increased fat mass and insulin resistance [16]. Furthermore, *miR‐148a* is required for the adipocyte differentiation and is highly increased in epididymal adipose tissue of mice fed HFD [17]. These results provide strong evidence for the miRNA pathway in regulating cellular energy metabolism in the adipose tissues.

Several studies have shown that tissue‐specific Ago2 and its mediated miRNA signaling pathways play a crucial role on body metabolism and contribute to the progression of obesity. However, it is unclear whether this function extends to other tissues, especially in adipose tissue. Therefore, this study will investigate the role of Ago2 in fat tissues. We find that HFD‐induced obesity is defined by the metabolic dysregulation and disrupted energy homeostasis, and that these changes are largely associated with the evaluated expression of Ago2 in different adipose depots. The increased Ago2 led to a concomitant upregulation of *miR‐148a* and downregulation in AMPK⍺ expression in adipose tissues. Finally, histological analysis and thermogenesis genes were performed to assess their relationships to HFD‐induced changes in miRNA signaling pathway. Together, those results demonstrate Ago2 as an important regulator in physiological processes of adipose tissues and further reinforce the role of Ago2‐mediated miRNA signaling in regulation of HFD‐induced obesity.

## Materials and methods

### Animals model

All animals were provided by the Laboratory Animal Center of the Xinxiang Medical University (Henan, China) and maintained on a 12 h light/dark cycle. To obtain HFD‐induced obesity model, C57/BL6N male mice (6 weeks old) were purchased from Beijing Vital River Laboratory (Beijing, China) and randomly assigned to two groups. Then, the mice were fed either normal chow diet (Chow) with 10 KJ% fat (Keao Xieli, Beijing) as control group, or HFD with 60 KJ% fat (Keao Xieli, Beijing) as obesity group *ad libitum* until 16 weeks. Before adipose tissue was collected, the mice were anesthetized with isoflurane and sacrificed after cervical dislocation. All animal experiments were carried out in accordance with Chinese Council on Animal Care guidelines, and the study was approved by the Animal Research Committee of Xinxiang Medical University (Approved ID XYLL‐2020158).

### Body weight and composition analysis

Body weights were measured every week with a digital scale. Body composition of lean body mass (LBM) and fat mass were measured using time‐domain nuclear magnetic resonance (TD‐NMR) method (the minispec Live Mice Analyzer LF90, Bruker, Beijing, China).

### Analysis of glucose metabolism in mice

Blood samples were collected from submandibular vein and centrifuged at 2000 ***g*** for 10 min. Plasma insulin and leptin were measured by commercial enzyme‐linked immunosorbent assay (ELISA) kit (Crystal Chem Inc. #90080, #90030, Elk Grove Village, IL, USA). Plasma triglyceride, free fat acid (FFA), glycerol, and cholesterol levels were measured using commercially available kits (Cayman, 10010303, 700310, 10010755, 10007640).

For glucose tolerance test (GTT), mice were injected intraperitoneally (i. p.) with D‐glucose (2 mg·g^−1^ body weight) after overnight fasting, blood glucose levels were measured using Glucometer (Contour, Bayer, Beijing, China) before and following the injection from the facial vein of nonanesthetized animals. For insulin tolerance test (ITT), 6 h fasted mice were injected by i.p. with human recombinant insulin (0.75 U·kg^−1^ body weight, PAN Biotech, Aidenbach, Germany), and tail‐tip blood glucose was measured before and after the injection.

### Metabolic phenotyping analysis

16‐week‐old chow diet‐fed mice and HFD‐fed mice were acclimated into home cage of Promethion Core System (Sable Systems International, Beijing, China) for 24 h before the start of the experimental record. VO_2_ and VCO_2_ level were measured for 1 min in a 9 min interval for 4 consecutive days and locomotor activity was measured continuously by breaks of light beams. O_2_ consumed (VO_2_), CO_2_ produced (VCO_2_), and energy expenditure (EE) were calculated following the manufacture's manual, and the respiratory exchange ratio (RER) was computed by the ratio of VCO_2_/VO_2_. Finally, EE was analyzed either after the normalization by LBM or locomotor activity using covariance (ANCOVA).

### Western blot

Total protein of brown adipose tissue (BAT), subcutaneous white adipose tissues (sWAT), and epididymal white adipose tissues (eWAT) were lysed by RIPA buffer, and quantitated by Pierce™ BCA Protein Assay kit (23225, Thermo Fisher Scientific, Beijing, China). Equal amounts of protein (20 μg) were separated by SDS/PAGE electrophoresis, transferred to PVDF membrane, and blocked in 5% skim milk in TBST. Then, the membrane was incubated overnight with primary antibody solution at 4 °C. After rinsing the blot, the membrane was incubated in the horseradish peroxidase (HRP)‐conjugated secondary antibody solution for 2 h at room temperature. Finally, chemiluminescent signals on the blot were applied with SuperSignal™ West Femto Maximum Sensitivity Substrate (34095, ThermoFisher Scientific), and captured using Amersham Imager 600 imagers (GE Healthcare Life Sciences, Tokyo, Japan). The band intensity of protein was read by image analysis software fiji . The following primary and secondary antibodies were used in this study: Ago2 (RN003M, MBL International, 1 : 1000, Tokyo, Japan), UCP1 (#14670, Cell Signaling Technology, 1 : 1000, Shanghai, China), AMPKα (#2532, Cell Signaling Technology, 1 : 1000), p‐AMPKα (#2535, Cell Signaling Technology, 1 : 1000), GAPDH (ab8245, Abcam, 1 : 1000), Goat Anti‐Rabbit IgG H&L (HRP) (ab6721, Abcam, 1 : 10,000, Shanghai, China), Goat Anti‐Mouse IgG H&L (HRP) (ab6789, Abcam, 1 : 10,000). The quantification of blots was analyzed by ImageJ (NIH, https://imagej.nih.gov/ij/). The raw images of western blots were seen in Figure  S1.

### Quantitative real‐time polymerase chain reaction (qPCR)

Total RNA was isolated from BAT, sWAT, eWAT, and inguinal white adipose tissue (ingWAT) using RNeasy plus Mini kit (QIAGEN, Hilden, Germany) and quantitated by Spark^®^ (TECAN, Shanghai, China). First strand cDNA from total RNA was efficiently synthesized using RevertAid First Strand cDNA Synthesis Kit (#K1621, ThermoFisher Scientific). Quantitation of *miR‐148a‐3p* expression level was performed with TaqMan™ MicroRNA Assay (4440887, Applied Biosystems, Beijing, China) and normalized to U6 snRNA following the primers from Applied Biosystems: *mmu‐miR‐148a‐3p* (mmu477814_mir) and U6 snRNA (001973). qPCR of mRNAs was detected using FastStart SYBR Green Master (04673484001, Roche, Shanghai, China) and normalized to β‐actin in StepOne™ Real‐Time PCR Systems (Applied Biosystems). The sets of primer sequence were ordered from ThermoFisher Scientific in Table S1.

### Cell culture

The HEK293T cell line was obtained from CCTCC (1101HUM‐PUMC000091, Beijing). Cells were cultured in Dulbecco's modified Eagle's medium (DMEM; 10569010, Gibco, Beijing, China) supplemented with 10% fetal bovine serum (FBS; abs972, Absin) and 1% penicillin/streptomycin (15140122, Gibco) in a 5% CO2 atmosphere at 37 °C.

### Luciferase assay and transfection

The 3′‐UTR of murine *AMPKα* was PCR amplified using the following primers 5′‐TGG TAG CAT AGC ATA ATG GG‐3′ and 5′ ‐CAA CAG TTT ATA GAG ATA TTC CTC AG‐3′ and cloned into the pGL3 Luciferase Reporter Vectors (Promega, Beijing, China). HEK293T cells were seeded for co‐transfection of *AMPKα* 3′‐UTR plasmid and *miR‐148a* mimics (MC10263, ThermoFisher Scientific) or *mir*Vana™ miRNA Mimic, Negative Control #1 (4464058, ThermoFisher Scientific). After 48 h of transfections, luciferase assays were detected using the Dual‐Luciferase^®^ Reporter Assay System (Promega) following the manufacturer's manual.

The pECMV vector carrying the full‐length cDNA of mouse Ago2 (pECMA‐Ago2‐m‐Flag) was purchased from Bionice Biotechnology Co. Ltd. (Ningbo, China). The plasmid was identified by sequencing (GENEWIZ, Suzhou, China). According to the manufacturer's protocol, pECMV‐Ago2 or control pECMV plasmids were transfected into HEK293T cells using Lipofectamine 3000 reagent (ThermoFisher Scientific). After 48 h of transfection, the expression of Ago2 was examined by qPCR and western blot.

### Histological detection

The adipose tissue pads and liver were dissected from 16‐week‐old mice, fixed in 4% paraformaldehyde (PFA), and embedded in paraffin. Tissue sections were stained with hematoxylin and eosin (H&E) and imaged using a Nikon ECLIPSE 80*i* microscope, Tokyo, Japan. The raw images of the microscope image were seen in Figure S2.

### Statistical analyses

Results were reported as mean ± SEM from at least three independent experiments and statistical analysis is summarized in Table S2. A *P*‐value of ≤ 0.05 was considered statistically significant (see Table S2). *P < 0.05, **P < 0.01, and ***P < 0.001. All graphical and statistical analyses were performed using the Prism9 software (Graphpad Software, San Diego, CA, USA), Microsoft Excel and SPSS. Comparisons between data sets with two groups were evaluated using an unpaired Student's *t* test. ANOVA analysis was performed for comparisons of three or more groups.

## Results

### High‐fat diet induces obesity

**Fig. 1:**
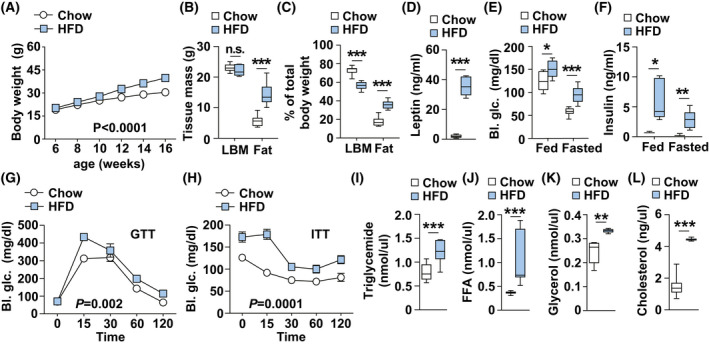
High‐ fat diet results in increased body weight and insulin resistance. (A) Body weight curves of HFD‐fed mice (*n* = 12) and normal chow diet‐fed controls from 6 to 16 weeks of age. (B) Lean body mass (LBM) and fat mass in 16‐week‐old HFD (*n* = 6) and normal chow diet‐fed mice (*n* = 11). (C) Tissue mass to body weight ratio in HFD (*n* = 6) and control mice (*n* = 11). (D) Plasma leptin measurements in 16‐week‐old HFD (*n* = 9) and chow diet controls (*n* = 10). (E, F) Random and fasted plasma glucose and insulin measurement of HFD (*n* = 6) and chow diet‐fed mice at 16 weeks (*n* = 11). (G, H) Glucose measurements during glucose tolerance test (GTT) (G) and insulin tolerance test (ITT) (H) on 12‐week‐old HFD (*n* = 5) and normal chow diet‐fed mice (*n* = 8). (I–L) Plasma triglyceride (chow: *n* = 11, HFD: *n* = 6), free fatty acid (FFA) (chow: *n* = 9, HFD: *n* = 9), glycerol (chow: *n* = 6, HFD: *n* = 6) and cholesterol measurement (chow: *n* = 10, HFD: *n* = 10) on 16‐week‐old HFD and normal chow diet‐fed mice. Results are presented as mean ± SEM. *P < 0.05, **P < 0.01 and ***P < 0.001.

To observe HFD‐induced obesity, C57/BL6J mice were divided into two groups and fed with normal chow diet or HFD from 6‐week‐old. Body weight was monitored weekly until the mice were 16‐week old. Interestingly, administration of the HFD can significantly increase in body weight compared to chow diet controls starting at 8‐week‐old age (Fig. 1A). The increased body weight was likely due to an increase in fat mass as shown by NMR analysis of body composition at 16 weeks of age (Fig. 1B,C). Consistent with increased adiposity, plasma levels of leptin were strongly increased almost 15‐fold in HFD‐fed mice compared to normal chow diet‐fed mice (Fig. 1D). Due to the association of obesity with diabetes and insulin resistance, we further analyzed the systemic glucose metabolism in HFD‐fed mice and normal chow diet‐fed mice. Both random and fasting plasma glucose and insulin levels were elevated in HFD‐fed mice (Fig. 1E,F). Glucose and insulin tolerance tests (GTT and ITT) showed higher glucose levels in HFD‐fed mice in comparison to chow diet‐fed controls indicating systemic insulin sensitivity was reduced (Fig. 1G,H). Lastly, the levels of plasma lipids including triglyceride, FFA, glycerol, and cholesterol were also significantly elevated in the HFD‐fed mice (Fig. 1I–L).

### Changes in basic metabolic rates in High‐fat diet feeding mice

**Fig. 2:**
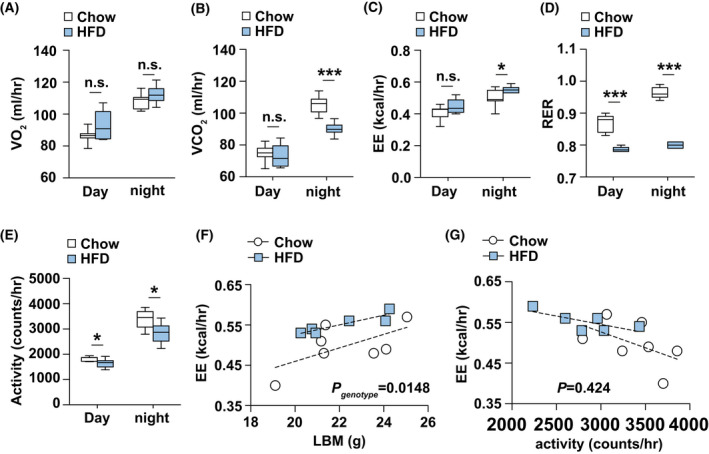
High‐fat diet alters energy metabolism in mice. (A–E) Quantification of O_2_ production, CO_2_ consumption, energy expenditure, RER (Respiratory exchange ratio) and locomotor activity, respectively, in 16‐week‐old HFD (*n* = 6) and chow diet‐fed mice (*n* = 7). (F) Energy expenditure of individual animals plotted against lean body mass from 16‐week‐old HFD (*n* = 6) and normal chow diet‐fed mice (*n* = 7). (G) Energy expenditure of individual animals plotted against locomotor activity from 16‐week‐old HFD (*n* = 6) and normal chow diet‐fed mice (*n* = 7). Results are presented as mean ± SEM. *P < 0.05, and ***P < 0.001.

To understand the factors which may contribute to the increase in body mass and adiposity, we next determined energy metabolism in these animals in HFD animals. Indirect gas calorimetry was used to calculate basic metabolic rates in these animals at 12‐week‐old age. As shown in Fig. 2, HFD‐fed mice exhibited no changes in oxygen consumption (Fig. 2A), but decreases in carbon dioxide production (Fig. 2B). Energy expenditure was slightly increased in HFD‐fed mice (Fig. 2C). These results translate into a decrease in the nutrient partitioning [based on respiratory exchange rate (RER)] measured in HFD‐fed mice and suggest fat to be the predominant energy source (Fig. 2D). Additionally, the spontaneous locomotor activity was reduced in HFD‐fed mice (Fig. 2E). Moreover, energy expenditure, when plotted in relation to lean body mass in an ANCOVA, is again increased in HFD‐fed mice (Fig. 2F). However, this difference disappears when energy expenditure is plotted in relation to locomotor activity (Fig. 2G). These results suggested that HFD animals of similar lean body mass to controls have increased energy expenditure is directly correlated to the shift in metabolic fuel from carbohydrates in normal chow diet to lipids in HFD.

### High‐fat diet increases Ago2 and *miR‐148a* expression in the adipose tissue

**Fig. 3:**
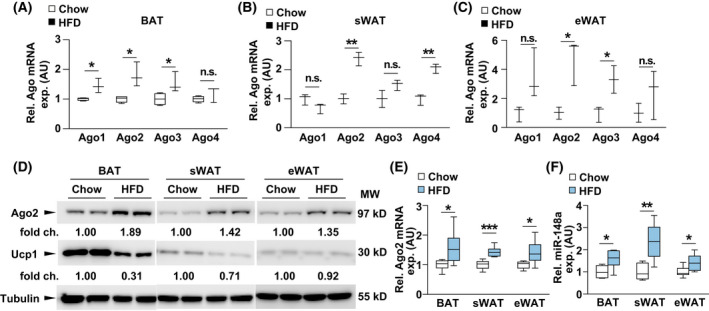
Increased expression of Ago2 and miR‐148a in fat tissues of HFD‐fed mice. (A–C) qPCR analysis of *Ago1‐4* from BAT, sWAT, and eWAT of HFD (*n* = 3) and chow diet‐fed mice (*n* = 3) at 16 weeks old. (D) Western blot analysis of Ago2 and Ucp1 in total lysate from BAT, sWAT, and eWAT of wild‐type mice on normal chow diet and littermate controls on HFD feeding. (E, F) qPCR analysis of *Ago2* and *miR‐148a* from BAT, sWAT, and eWAT of HFD (*n* = 6) and chow diet‐fed mice (*n* = 6) at 16 weeks old. Results are presented as mean ± SEM. *P < 0.05, **P < 0.01 and ***P < 0.001.

The Argonaute family of RNA‐binding proteins (Ago1‐4) are essential regulators of microRNA function. Due to miRNA dysregulation in the large repertoire of physiological processes of obesity [19], we sought to examine the expression of Ago family in obese adipose tissue. qPCR results indicated that Ago1 mRNA was only increased in the BAT of HFD‐fed mice (Fig. 3A–C); Ago3 was increased in both BAT and eWAT of HFD‐fed mice, but not in sWAT (Fig. 3A–C); Ago4 mRNA was elevated in the eWAT of HFD‐fed mice (Fig. 3A–C). Interestingly, the expression of Ago2, which has an endoribonuclease (slicer) activity, is found to be increased in all obese adipose tissues (Fig. 3A–C). Recent studies have revealed that Ago2‐miRNA‐mediated mRNA regulation plays a significant role in the hepatic and adipose energy metabolism of obesity [11]. Western blot analysis indicated an increased Ago2 protein levels in adipose tissue of HFD‐fed mice compared to normal chow diet‐fed control mice (Fig. 3D). Consistent with increased body fat mass, *Ucp1* expression is reduced in BAT and subcutaneous sWAT of HFD‐fed mice (Fig. 3D). Meanwhile, qPCR analysis further indicated increased *Ago2* mRNA in different fat tissues after HFD feeding (Fig. 3E). As *miR‐148a* has been involved in the energy metabolism [10], the expression level of *miR‐148a* was checked by qPCR. Consistent with the *Ago2*, *miR‐148a* expression levels were significantly increased in BAT, sWAT, and eWAT of HFD‐fed mice compared to normal chow diet‐fed control mice (Fig. 3F), these results support the critical role of Ago2 in the expression of *miR‐148a*.

### Ago2 regulates miRNA‐mediated targeting of *AMPKα*

**Fig. 4:**
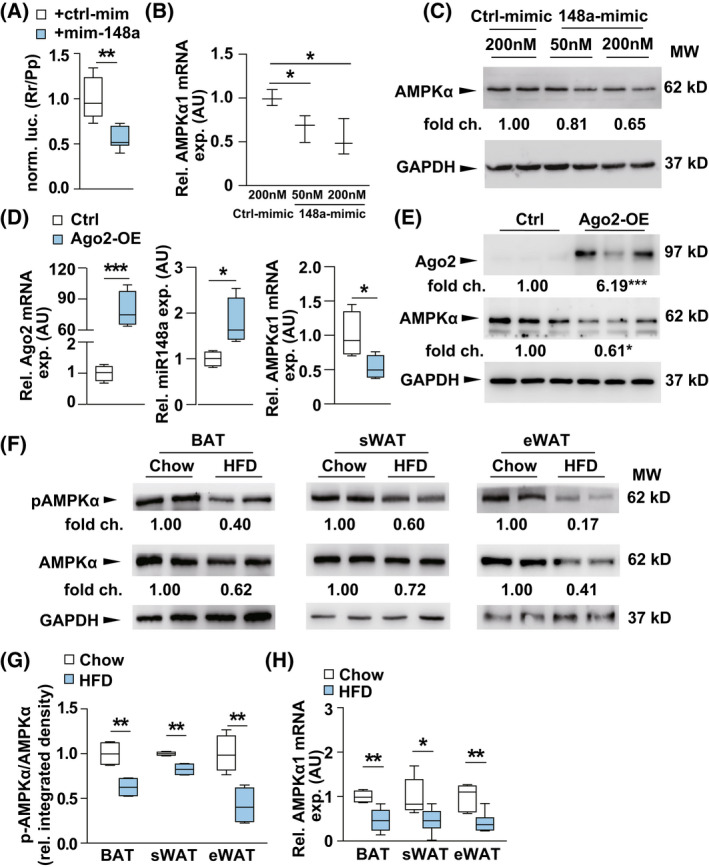
Ago2 regulates miRNA‐mediated targeting of AMPKα. (A) Luciferase assays in HEK293 cells testing direct targeting of AMPKα genes by miR‐148a (148a‐mimic) (*n* = 6). (B) qPCR analysis of *AMPKα1* in HEK293 cells after transfection of *miR‐148a* and *control mimic* (*n* = 3). (C) Western blot analysis of AMPKα in HEK293 cells after transfection of *miR‐148a* and *control mimic*. (D), qPCR analysis of *Ago2*, *miR‐148a*, *AMPKα1* in HEK293 cells after transfection of pECMV‐Ago2 (Ago2‐OE) (*n* = 4) and an empty vector pECMV (control) (*n* = 4). (E) Western blot analysis of Ago2 and AMPKα in HEK293 cells after transfection of pECMV3‐Ago2 (Ago2‐OE) and an empty vector pECMV3 (control). (F) Western blot analysis of p‐AMPKα and AMPKα from BAT, sWAT, and eWAT of HFD and chow diet‐fed mice at 16 weeks. (G) Quantification of p‐AMPKα/AMPKα from BAT, sWAT, and eWAT of HFD (*n* = 4) and chow diet‐fed mice (*n* = 4) at 16 weeks old. (H) qPCR analysis of *AMPKα1* from BAT, sWAT, and eWAT of HFD (*n* = 6) and chow diet‐fed (*n* = 6) mice at 16 weeks old. Results are presented as mean ± SEM. *P < 0.05, **P < 0.01, and ***P < 0.001.

To elucidate the molecular basis for the role of Ago2 in adipose tissues, we next sought to identify candidate target genes that are involved in the lipid metabolism. We first hypothesized that AMPKα may be subject to Ago2: miRNA‐mediated gene regulation in the adipose tissue in light of its prominent role in lipid metabolism as well as its establish role as an energy and metabolic sensor in response to changes in nutrient and hormone availability [20, 21]. Importantly, *AMPKα*'s 3′ UTR has been reported to have an *miR‐148/152* target site [10]. To establish the direct interaction between Ago2, *miR‐148a*, and the *AMPKα* mRNA, we performed luciferase assays in which *AMPKα*'s 3′ UTR was cloned into luciferase expression vector. The luciferase activity of each was measured in HEK293 cells. Comparing to control miRNA mimics, *miR‐148a* mimics can strongly inhibit the luciferase activity (Fig. 4A). Both Western blots and qPCR indicated a strongly decreased protein and mRNA level of AMPKα after transfection of *miR‐148a* mimics (Fig. 4B,C).

To experimentally assess whether Ago2 is a physiologically relevant regulator of AMPKα, gain‐of‐function approach using the plasmid encoding mouse *Ago2* full length cDNA was performed in the HEK cells. Over‐expression (Ago2‐OE) conditions were validated by immunoblotting and qPCR (Fig. 4D,E). Consistent with results from HFD‐fed mice (Fig. 3), over‐expression of Ago2 can significantly increase *miR‐148a* level (Fig. 4D) and subsequently inhibit both mRNA and protein expression of AMPKα (Fig. 4D–E). We next measured expression of *AMPKα* in different adipose tissues. Western blot confirmed a similar decrease in both total AMPKα protein and p‐AMPKα in HFD‐fed mice compared to normal chow diet‐fed control mice (Fig. 4F), which is associated with a decreased AMPK activity represented as the ratio of p‐AMPKα/AMPKα in the fat tissues of HFD‐fed mice (Fig. 4G). qPCR also identified the down‐regulation of *AMPKα* mRNA after HFD feeding (Fig. 4H). Together, those data indicated that *miR‐148a* is involved in suppression of *AMPKα* expression in a manner dependent on Ago2 and a *miR‐148a* target site.

### High‐fat diet causes the lipid accumulation by changing lipid metabolism

**Fig. 5:**
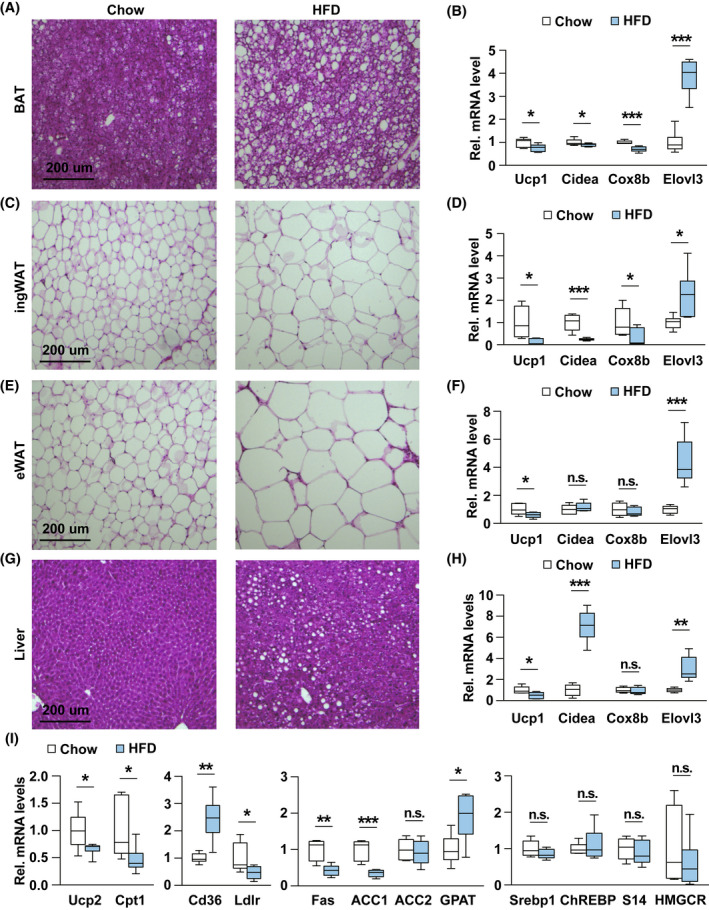
Lipid accumulation in HFD‐fed mice. (A–H) Hematoxilin and eosin staining of BAT, ingWAT, eWAT, liver, and qPCR gene expression analysis from 16‐week‐old normal chow diet‐fed (*n* = 6) and HFD‐fed mice (*n* = 6). (I) qPCR analysis for gene expression from liver of 16‐week‐old normal chow diet (*n* = 6)‐ and HFD‐fed mice (*n* = 6). Scale bar = 200 μm (A, C, E, G). Results are presented as mean ± SEM. *P < 0.05, **P < 0.01, and ***P < 0.001.

To further determine the causes for the increased body weight, we next performed histological analysis in the BAT, ingWAT, eWAT, and liver. As expected, hematoxylin and eosin stain revealed a massive lipid accumulation in HFD fed BAT compared to normal chow diet‐fed mice (Fig. 5A). Consistent with this observation, gene expression analysis showed HFD increased *Elovl3* expression, which is involved in fat cell elongation, but decreased expression of genes involved in BAT thermogenesis and oxidation (including *Ucp*1, *Cidea*, *Cox8b*) (Fig. 5B). Histological analysis of WAT tissue suggests an adipocyte hypertrophy in the adipose tissue of HFD‐fed mice (Fig. 5C,E), and gene expression analysis showed significantly increased expression of *Elovl3* of HFD‐fed mice (Fig. 5D,F). Furthermore, expression analysis in ingWAT of BAT‐enriched genes was lower notably in HFD‐fed mice compared with chow diet‐fed mice (Fig. 5D), but in eWAT was unaltered (Fig. 5F). These data suggest that HFD leaded to the loss of beige fat cells in ingWAT, which almost exists exclusively in subcutaneous depots. Finally, image analysis of liver also shows strongly increased lipid accumulation in liver (Fig. 5G). Consistently, the gene expression of *Cidea* increased significantly in liver (Fig. 5H). Also, the expression of β‐oxidation‐related genes in mitochondria such as *UCP1*, *Ucp2*, and *Cpt1* decreased (Fig. 5H,I). The increased expression of *Elovl3*, *Cpt1*, and Cd36 suggested increased synthesis of fatty acids and triglyceride, and free fatty acid uptake, respectively (Fig. 5H,I). The expression of genes related to *de novo* fatty acids synthesis such as *Fas*, and *ACC1* showed decreased expression (Fig. 5I), which implicated the hepatic lipid accumulation induced by HFD was not caused by the increase of *de novo* fatty acids synthesis, but more likely due to the ectopic lipid deposition triggered by the excessive fat storage in adipose tissue.

## Discussion

Numerous studies have described mechanisms which illustrate the contribution of the miRNA signaling in maintaining the balance between energy intake and expenditure. In this study, we show that mice feeding with HFD exhibit diet‐induced obesity and decreases in glucose homeostasis, insulin sensitivity, and energy expenditure rate. Mechanistically, our measurements further show HFD induced obesity resulted in an alteration in expressions of Ago2‐mediated miRNA signaling in adipose tissues. This causes suppression of miR‐148a target gene *AMPKα*, which is an important nutrient and energy sensor in maintaining energy homeostasis. Those results are in line with previous studies showing Ago2 and miRNAs are involved adipogenesis [16, 22] and emphasize an important role for Ago2 in regulation of body homeostasis and fat adiposity.

Functionally, Ago2 is expressed in a number of tissues including the liver and pancreas, and is critical for the obesity‐associated pathophysiology [8, 10]. For example, in pancreatic β cells, the expression of Ago2 is significantly increased in the *ob/ob* islets and shows beneficial impacts on obesity and insulin resistance by promoting pancreatic β cell expansion [8]. Notably, Ago2 is also abundant in the liver cells plays a crucial role in the regulation of glucose homeostasis in severe obesity [9, 10]; Ago2‐deficiency in the liver could protect against severe obesity‐induced insulin resistance and diabetes via Ago2‐PPARα signaling pathway [11, 12]. Now, our study further provides evidence that Ago2 is expressed in adipose tissues and it's expression is increased in diet‐induced model of obesity, in consistent with the role of Ago2 in obesity. Those data indicated that Ago2 protein is important in the metabolic regulation of different organs during obesity.

Furthermore, recent studies showed that several miRNAs controlled by Ago2 are key regulator of adiposity and involved in biological functions of adipogenesis. For example, *miR‐148a* is a key miRNA and its expression gradually increased in the process of controlling human adipose‐derived mesenchymal stem cells differentiation into adipocytes through binding to the target gene *WNT1* [17, 22]. Loss of *miR‐93* can increase adipose cell differentiation, fat mass, and subsequently insulin resistance by targeting *Tbx3* gene [16]. Furthermore, *miR‐34a* has been found to be increased which is associated with obesity and inhibit fat cell browning; Downregulation of *miR‐34a* can elevate expression of *FGF21* and results in induction of the browning genes *Ucp1*, *Pgc‐1*, and *Prdm16* [23]. Consistent with above studies is our observation showing an increase in expression of *miR‐148a* in the adipose tissues of HFD‐fed mice.

A common characteristic of obesity is high circulating lipid levels, partly accounted by impaired insulin‐mediated suppression of lipolysis. AMPKα, an important cellular energy sensor, has been implicated in control of whole‐body adiposity through regulation of lipolysis [24]. For example, AMPK inhibits lipogenesis in isolated adipocytes via increased ACC phosphorylation in response to AICAR stimulation [25], while enhanced activation of AMPK can promote lipolysis [26]. While knockout of AMPKα in mice caused an increased body weight and fat mass due to the enlarged adipocytes and lipid accumulation in fat cells [27]. Consequently, we speculated that the regulation of adipose tissue by Ago2 is realized through Ago2‐*miR‐148a*‐*AMPKα* signaling pathway. Obesity induces the increase of Ago2 expression, which enhances the silencing effect of *miR‐148a* on *AMPKα*, and subsequently reduces the activity of *AMPKα*, inhibits the calorie burning ability of adipocytes, and finally leads to lipid accumulation.

## Conclusion

In conclusion, our observations on the Ago2 provide further insight into identifying a specific miRNA signaling which regulate obesity and energy homeostasis in adipose tissue. Improving our understanding of Ago2 in adiposity and lipid metabolism may facilitate mapping of distinct systemic networks to delineate the unique properties of separate cell populations contributing to metabolism. It will be important to further investigate mechanism of how Ago2 and related miRNAs in the progression of diet‐induced obesity.

## Conflict of interest

The authors declare no conflict of interests.

## Author contributions

HZ, LQ, XL, XH, and JK performed the expression analysis, animal experiments, microscopy image analysis, and morphometric analysis. YL performed statistical analysis. LQ, JL and XY conceived and designed the study and wrote the manuscript. All authors contributed to interpretation of the data and approved the final version of this manuscript.

## Supporting information


**Fig. S1.** Scans of raw western blots shown in Figs 3 and 4.

**Fig. S2.** Scans of raw images shown in Fig. 5.

**Table S1.** The primer sequence of qPCR.

**Table S2.** Summary of statistical analyses.

## Data Availability

All data generated or analyzed during this study are included in this published article (and its Supplementary Information files) and are available from the corresponding author on reasonable request.
