# Supplementary figures and images for: Differential expression of Ago2‐mediated microRNA signaling in adipose tissue is associated with food‐induced obesity
Source: FEBS Open Bio. 2022 Sep 5;12(10):1828–38. doi: 10.1002/2211-5463.13471 (PMC9527595; doi:10.1002/2211-5463.13471)

Fig.3D

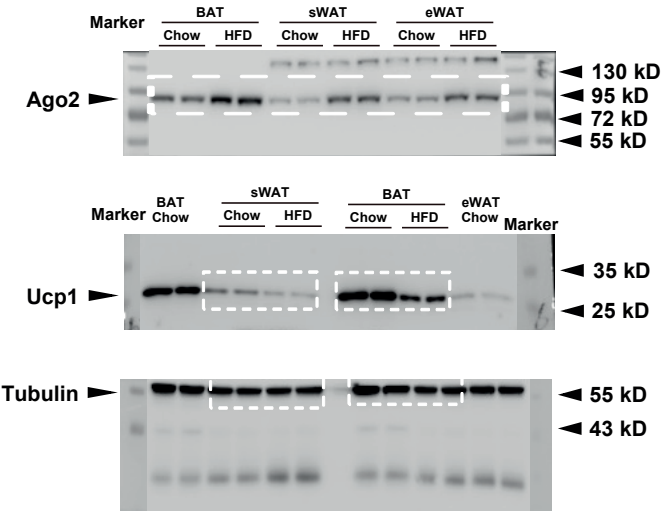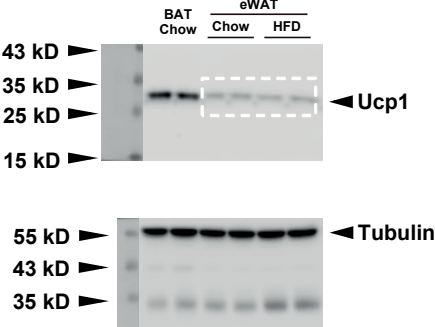

Fig.4C

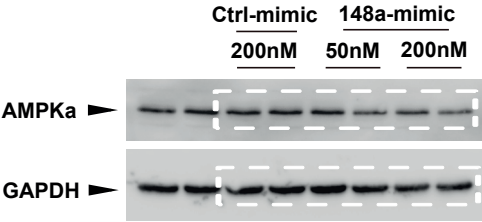

Fig. 4E

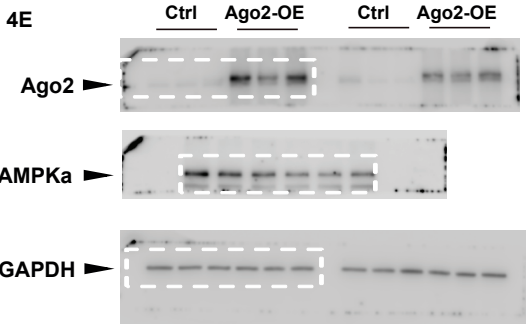

Fig.4F

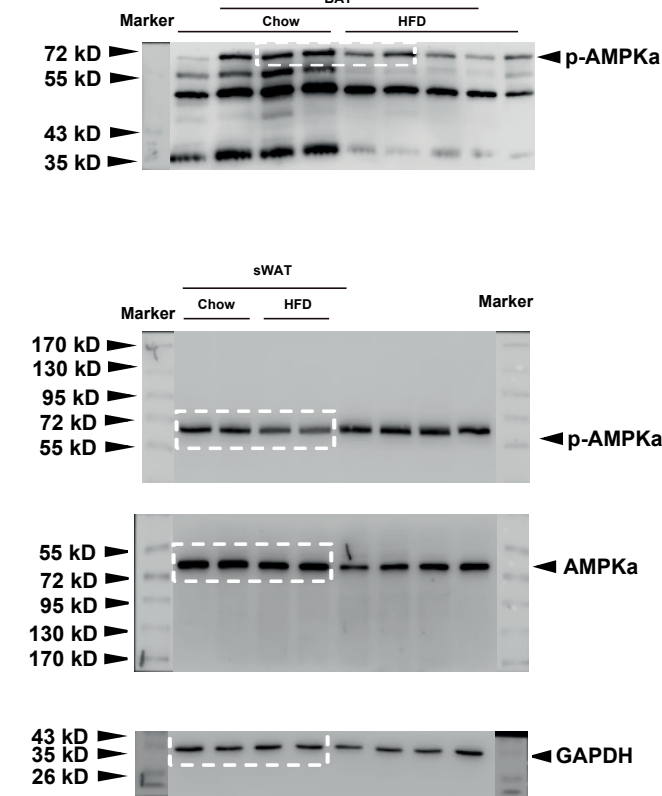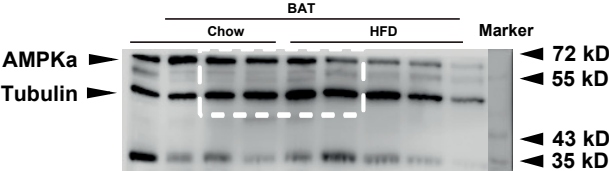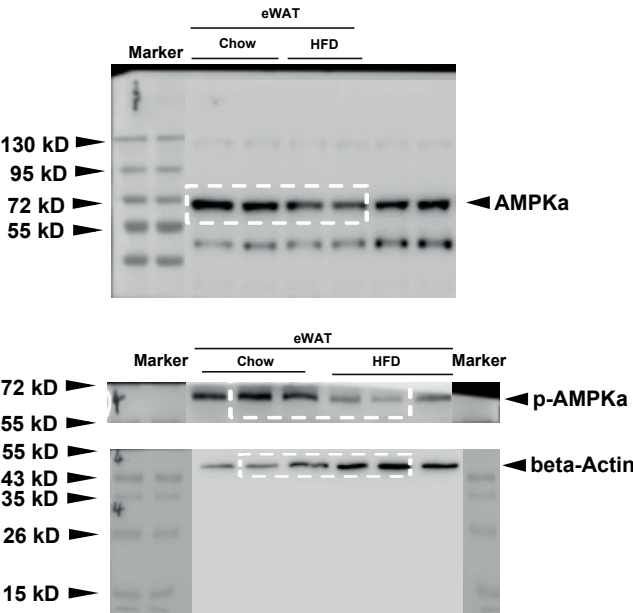

Supplement: Supplementary file 1 — Fig. S1. Scans of raw western blots shown in Figs 3 and 4. [file FEB4-12-1828-s003.pdf]

**Fig.5A**

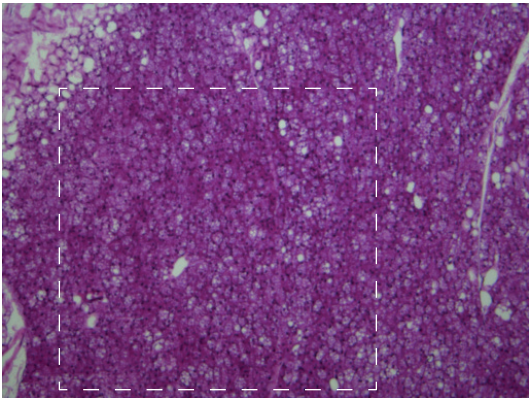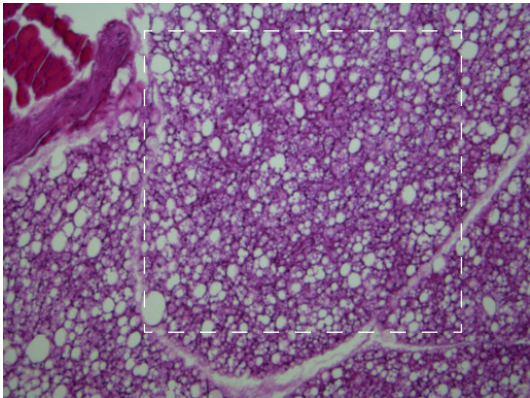

**Fig.5C**

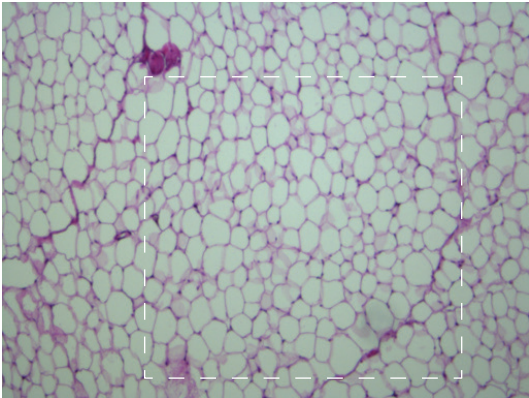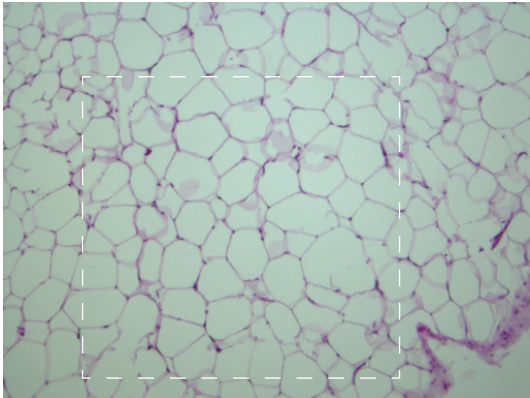

**Fig.5E**

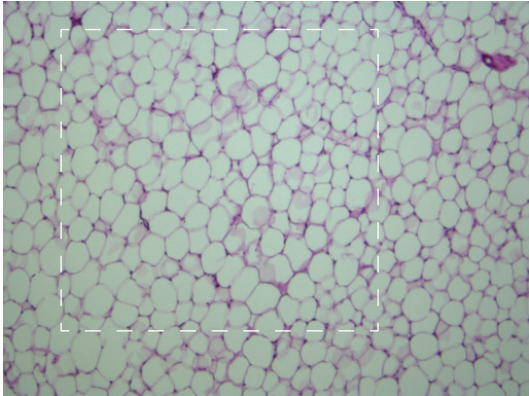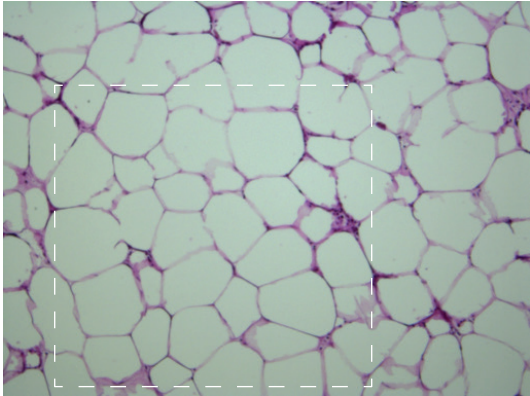

**Fig.5G**

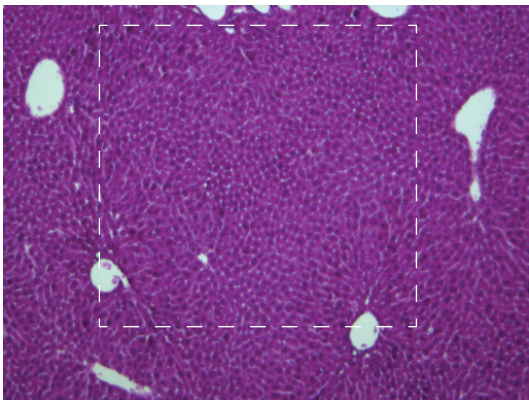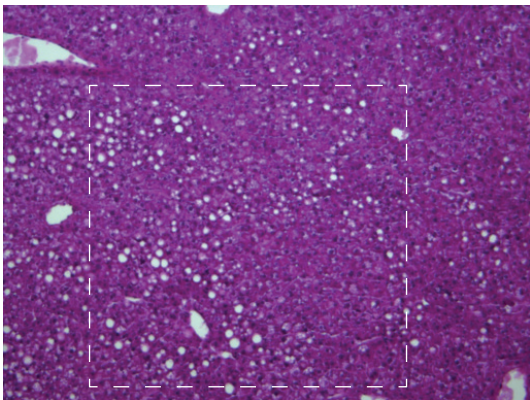

**Note:** Dashed white lines identify cropped areas

Supplement: Supplementary file 2 — Fig. S2. Scans of raw images shown in Fig. 5. [file FEB4-12-1828-s004.pdf]
