# Supplementary material for: Differential expression of Ago2‐mediated microRNA signaling in adipose tissue is associated with food‐induced obesity
Source: FEBS Open Bio. 2022 Sep 5;12(10):1828–38. doi: 10.1002/2211-5463.13471 (PMC9527595; doi:10.1002/2211-5463.13471)
Supplement: Supplementary file 3 — Table S1. The primer sequence of qPCR. [file FEB4-12-1828-s001.pdf]

## Supplementary Table S1 The primer sequence of mRNAs for qPCR

| Genes | GenBank™ accession no. | Sequence (5′-3′) |
|---|---|---|
| *β-actin* | NM_007393 | Forward: GTCCTCTCCCAAGTCCACAC<br>Reverse: GGGAGACCAAAAGCCTTCAT |
| *Ago2* | NM_153178 | Forward: GCACTTACCATCCATGAGGTAC<br>Reverse: AAAGAGAAGGTCGGACGGACTGAT |
| *AMPKα1* | NM_001355640 | Forward: GGGATCCATCAGCAACTATCG<br>Reverse: GGGAGGTCACGGATCAGG |
| *Ucp1* | NM_009463 | Forward: AGGCTTCCAGTACCATTAGGT<br>Reverse: CTGAGTGAGGCAAAGCTGATTT |
| *Cidea* | NM_007702 | Forward: TGCTCTTCTGTATCGCCCAGT<br>Reverse: GCCGTGTTAAGGAATCTGCTG |
| *Cox8b* | NM_007751 | Forward: GAACCATGAAGCCAACGACT<br>Reverse: GCGAAGTTCACAGTGGTTCC |
| *Elovl3* | NM_007703 | Forward: CGTAGTCAGATTCTGGTCCT<br>Reverse: CCAGAAGAAGTGTTCCGTTG |
| *Ucp2* | NM_011671 | Forward: CTGCAGATCCAAGGGGAGAGT<br>Reverse: CCTTGGTGTAGAACTGTTTGACAG |
| *Cpt1* | NM_001039657 | Forward: CTCTCGACGAGCCAAACCC<br>Reverse: CCGCAATGATATACGAAACGC |
| *Cd36* | NM_001159558 | Forward: GAACCACTGCTTTCAAAAACTGG<br>Reverse: TGCTGTTCTTTGCCACGTCA |
| *Ldlr* | NM_010700 | Forward: TGACTCAGACGAACAAGGCTG<br>Reverse: CTAACTAAACACCAGACAGAGGC |
| *Fas* | NM_004104 | Forward: GGTTACACTGTGCTAGGTGTTG<br>Reverse: TCCAGGCGCATGAGGCTCAGC |
| *ACC1* | NM_133360 | Forward: AAGGGCTGCCTCTAATG<br>Reverse: GATGTAAGCGCCGAACT |
| *ACC2* | NM_133904 | Forward: GCCAGAAGCCCCCAAGAAAC<br>Reverse: CGACATGCTCGGCCTCATAG |
| *GPAT* | NM_001079096 | Forward: GACGTCCGTAAGAGCATGTTTTG<br>Reverse: CTTCACTTTGTTGGTGGATTTGG |
| *Srebp1* | NM_0011480 | Forward: TGAAGCCGGAGGTGGTAGA<br>Reverse: CTTCATCTGGCTGTCCTCAAAA |
| *ChREBP* | NM_001359237 | Forward: GTATGTGGAGCGGAGGAAGAG<br>Reverse: ATGACGGCCTCGGGTTT |
| *S14* | NM_009381 | Forward: TGAGAACGACGCTGCTGAAAC<br>Reverse: AGGTGGGTAAGGATGTGATGGAG |
| *HMGCR* | NM_008255 | Forward: CTCAGTTCAAATTCACAGGATGAAGTAAG<br>Reverse: ACGGCTTTCACGAGAAAGCTCTAG |
