# Supplementary material for: Differential expression of Ago2‐mediated microRNA signaling in adipose tissue is associated with food‐induced obesity
Source: FEBS Open Bio. 2022 Sep 5;12(10):1828–38. doi: 10.1002/2211-5463.13471 (PMC9527595; doi:10.1002/2211-5463.13471)
Supplement: Supplementary file 4 — Table S2. Summary of statistical analyses. [file FEB4-12-1828-s002.pdf]

Supplementary Table 2. Summary of statistical analyses

Figure	Sample size (n)	Statistical Test	Values
**1A**	Chow: n=11<br>HFD: n=12	Two-way repeated-measure ANOVA<br>Post-hoc multiple comparisons test (Sidak's)	Interaction: F=35.92 P=0.0468<br>Time: F=456.3, P<0.0001<br>Genotype: F=26.57, P<0.10001<br>Multiple comparison:<br>6 weeks: P=0.7823<br>8 weeks: P=0.1847<br>10 weeks: P=0.0663<br>12 weeks: P=0.0001<br>14 weeks: P<0.0001<br>16 weeks: P<0.0001
**1B**	Chow: n=11<br>HFD: n=6	Two-tailed unpaired Student's t-test	LBM: t=1.338, P=0.2009<br>Fat: t=6.743, P<0.0001
**1C**	Chow: n=11<br>HFD: n=6	Two-tailed unpaired Student's t-test	LBM: t=8.122, P<0.0001<br>Fat: t=8.331, P<0.0001
**1D**	Chow: n=10<br>HFD: n=9	Two-tailed unpaired Student's t-test	t=17.75, P<0.0001
**1E**	Chow: n=11<br>HFD: n=6	Two-tailed unpaired Student's t-test	Fed: t=2.563, P=0.0264<br>Fasted: t=6.285, P<0.0001
**1F**	Chow: n=11<br>HFD: n=6	Two-tailed unpaired Student's t-test	Fed: t=2.587, P=0.0361<br>Fasted: t=3.202, P=0.0084
**1G**	WT: n=8<br>HFD: n=5	Two-way repeated-measure ANOVA<br>Post-hoc multiple comparisons test (Sidak's)	Interaction: F=5.108, P=0.0018<br>Time: F=215, P<0.0001<br>Genotype: F=12.86, P=0.0043<br>Multiple comparison:<br>0 min: P>0.9999<br>15 min: P<0.0001<br>30 min: P=0.3461<br>60 min: P=0.0866<br>120 min: P=0.1487
**1H**	WT: n=5<br>HFD: n=8	Two-way repeated-measure ANOVA<br>Post-hoc multiple comparisons test (Sidak's)	Interaction: F=8.484, P<0.0001<br>Time: F=47.88, P<0.0001<br>Genotype: F=17.74, P=0.0015<br>Multiple comparison:<br>0 min: P=0.0043<br>15 min: P<0.0001<br>30 min: P=0.1302<br>60 min: P=0.1718<br>120 min: P=0.0184
**1I**	Chow: n=11<br>HFD: n=6	Two-tailed unpaired Student's t-test	t=4.347, P=0.0006
**1J**	Chow: n=9<br>HFD: n=9	Two-tailed unpaired Student's t-test	t=4.073, P=0.0009
**1K**	Chow: n=6<br>HFD: n=6	Two-tailed unpaired Student's t-test	t=4.529, P=0.0011
**1L**	Chow: n=10<br>HFD: n=10	Two-tailed unpaired Student's t-test	t=14.36, P<0.0001
**2A**	Chow: n=7<br>HFD: n=6	Two-tailed paired Student's t-test	Day: t=1.667, P=0.1237<br>Night: t=1.406, P=0.1873
**2B**	Chow: n=7<br>HFD: n=6	Two-tailed paired Student's t-test	Day: t=0.5561, P=0.5893<br>Night: t=5.575, P=0.0002
**2C**	Chow: n=7<br>HFD: n=6	Two-tailed paired Student's t-test	Day: t=1.437, P=0.1785<br>Night: t=2.242, P=0.0466
**2D**	Chow: n=7<br>HFD: n=6	Two-tailed paired Student's t-test	Day: t=7.016, P<0.0001<br>Night: t=19.31, P<0.0001
**2E**	Chow: n=7<br>HFD: n=6	Two-tailed paired Student's t-test	Day: t=2.235, P=0.0471<br>Night: t=2.489, P=0.0301
**2F**	Chow: n=7<br>HFD: n=6	Linear regression	F=7.935, $P_{genotype}$=0.01483
**2G**	WT: n=7<br>HFD: n=6	Linear regression	F=5.095, P=0.5678
**3A**	Chow: n=3<br>HFD: n=3	Two-tailed unpaired Student's t-test	Ago1: t=3.711, P=0.0288<br>Ago2: t=3.852, P=0.012<br>Ago3: t=2.649, P=0.0455<br>Ago4: t=1.367, P=0.272
**3B**	Chow: n=3<br>HFD: n=3	Two-tailed unpaired Student's t-test	Ago1: t=2.018, P=0.1137<br>Ago2: t=8.292, P=0.0012<br>Ago3: t=2.428, P=0.0722<br>Ago4: t=7.133, P=0.002
**3C**	Chow: n=3<br>HFD: n=3	Two-tailed unpaired Student's t-test	Ago1: t=2.36, P=0.0777<br>Ago2: t=3.917, P=0.0173<br>Ago3: t=3.618, P=0.0224<br>Ago4: t=1.333, P=0.2534

**3E**	Chow: n=6<br>HFD: n=6	Two-tailed unpaired Student's t-test	BAT: t=2.373, P=0.0391<br>ingWAT: t=4.596, P=0.0010<br>eWAT: t=2.398, P=0.0374
**3F**	Chow: n=6<br>HFD: n=6	Two-tailed unpaired Student's t-test	BAT: t=2.753, P=0.0204<br>ingWAT: t=3.756, P=0.0037<br>eWAT: t=2.266, P=0.0469
**4A**	ctrl-mim: n=6<br>mim-148a: n=6	Two-tailed unpaired Student's t-test	t=4.115, P=0.0021
**4B**	ctrl-mim: n=3<br>50nM-mim-148a: n=3<br>200nM-mim-148a: n=3	Two-tailed unpaired Student's t-test	50nM-mim-148a: t=3.285, P=0.0304<br>200nM-mim-148a: t=3.551, P=0.0238
**4D**	Ctrl: n=4<br>Ago2-OE: n=4	Two-tailed unpaired Student's t-test	Ago2: t=8.919, P=0.0001<br>miR148: t=2.94, P=0.026<br>AMPK: t=2.499, P=0.0466
**4E**	Ctrl: n=3<br>Ago2-OE: n=3	Two-tailed unpaired Student's t-test	Ago2: t=9.584, P=0.0007<br>AMPK: t=4.828, P=0.0085
**4G**	Chow: n=4<br>HFD: n=4	Two-tailed unpaired Student's t-test	BAT: t=4.264, P=0.0053<br>sWAT: t=4.671, P=0.0034<br>eWAT: t=3.998, P=0.0071
**4H**	Chow: n=6<br>HFD: n=6	Two-tailed unpaired Student's t-test	BAT: t=4.328, P=0.0015<br>sWAT: t=2.695, P=0.0225<br>eWAT: t=3.83, P=0.0033
**5B**	Chow: n=6<br>HFD: n=6	Two-tailed unpaired Student's t-test	Ucp1: t=2.27, P=0.0466<br>Cidea: t=2.413, P=0.0365<br>Cox8b: t=5.187, P=0.0004<br>Elov13: t=7.844, P<0.0001
**5D**	Chow: n=6<br>HFD: n=6	Two-tailed unpaired Student's t-test	Ucp1: t=3.072, P=0.0118<br>Cidea: t=4.992, P=0.0005<br>Cox8b: t=2.268, P=0.0467<br>Elov13: t=2.838, P=0.0176
**5F**	Chow: n=6<br>HFD: n=6	Two-tailed unpaired Student's t-test	Ucp1: t=2.251, P=0.0481<br>Cidea: t=0.8966, P=0.3910<br>Cox8b: t=0.789, P=0.4484<br>Elov13: t=4.92, P=0.0006
**5H**	Chow: n=6<br>HFD: n=6	Two-tailed unpaired Student's t-test	Ucp1: t=2.788, P=0.0192<br>Cidea: t=9.539, P<0.0001<br>Cox8b: t=0.5525, P=0.5927<br>Elov13: t=4.18, P=0.0019
**5I**	Chow: n=6<br>HFD: n=6	Two-tailed unpaired Student's t-test	Ucp2: t=2.287, P=0.0453<br>Cpt1: t=2.249, P=0.0482<br>Cd36: t=4.422, P=0.0013<br>Ldlr: t=2.229, P=0.0499<br>Fas: t=4.316, P=0.001522<br>ACC1: t=5.426, P=0.00029<br>ACC2: t=0.4785, P=0.642607<br>GPAT: t=2.93, P=0.01504<br>Srebp1: t=1.475, P=0.170981<br>ChREBP: t=0.5828, P=0.572914<br>S14: t=0.6546, P=0.527494<br>HMGCR: t=0.7004, P=0.5035
